# Microchromosome BAC-FISH Reveals Different Patterns of Genome Organization in Three Charadriiformes Species

**DOI:** 10.3390/ani12213052

**Published:** 2022-11-06

**Authors:** Marcelo Santos de Souza, Suziane Alves Barcellos, Michelly da Silva dos Santos, Ricardo José Gunski, Analía del Valle Garnero, Edivaldo Herculano Corrêa de Oliveira, Rebecca E. O’Connor, Darren K. Griffin, Rafael Kretschmer

**Affiliations:** 1 Laboratório de Diversidade Genética Animal, Universidade Federal do Pampa, São Gabriel 97300-162, RS, Brazil; 2 Laboratório de Cultura de Tecidos e Citogenética, SAMAM, Instituto Evandro Chagas, Ananindeua 67030-000, PA, Brazil; 3 Instituto de Ciências Exatas e Naturais, Universidade Federal do Pará, Belém 66075-110, PA, Brazil; 4 School of Biosciences, University of Kent, Canterbury CT2 7NJ, UK; 5 Departamento de Ecologia, Zoologia e Genética, Instituto de Biologia, Universidade Federal de Pelotas, Pelotas 96010-900, RS, Brazil

**Keywords:** microchromosome, avian karyotype, bird, BAC, FISH, comparative genomics, molecular cytogenetics

## Abstract

**Simple Summary:**

Numerous tiny (micro)chromosomes are a characteristic feature associated with birds, being found in smaller numbers in other organisms and absent in many, such as mammals. Although microchromosomes constitute a large portion of the genome in birds, data on them pertaining to comparative studies between birds are still scarce. This is the case in shorebirds (Charadriiformes), a group with a great variety of species. The aim of this study was to provide insight regarding the evolution of the microchromosomes of three species of shorebirds—the red knot (*Calidris canutus*), the wattled jacana (*Jacana jacana*), and the southern lapwing (*Vanellus chilensis*). The experiments are referred to as cross-species fluorescence in situ hybridization (FISH) mapping using probes called bacterial artificial chromosomes (or BACs), two (one labelled in red and one labelled in green) for every microchromosome. The results thus appear as the microchrochromosome with one green and one red end, revealing different patterns of organization over evolutionary time. In the red knot, they fuse together, but in the southern lapwing, they hardly change. We also described a new chromosome number for the red knot (92 in total). In conclusion, this study contributed to the understanding of microchromosomes organization and evolution of three shorebird species.

**Abstract:**

Microchromosomes, once considered unimportant elements of the genome, represent fundamental building blocks of bird karyotypes. Shorebirds (Charadriiformes) comprise a wide variety of approximately 390 species and are considered a valuable model group for biological studies. Despite this variety, cytogenetic analysis is still very scarce in this bird order. Thus, the aim of this study was to provide insight into the Charadriiformes karyotype, with emphasis on microchromosome evolution in three species of shorebirds—*Calidris canutus*, *Jacana jacana*, and *Vanellus chilensis*—combining classical and molecular approaches. Cross-species FISH mapping applied two BAC probes for each microchromosome, GGA10–28 (except GGA16). The experiments revealed different patterns of microchromosome organization in the species investigated. Hence, while in *C*. *canutus,* we found two microchromosomes involved in chromosome fusions, they were present as single pairs in *V*. *chilensis*. We also described a new chromosome number for *C*. *canutus* (2n = 92). Hence, this study contributed to the understanding of genome organization and evolution of three shorebird species.

## 1. Introduction

The order Charadriiformes, commonly known as shorebirds, comprises approximately 390 species, divided into 13 families [1]. According to Baker et al. [2], the morphological analysis of the fossils with molecular studies suggests that this group originated during the Cretaceous period between 79 and 102 million years ago. Phylogenetic studies support three major clades: Lari (gulls, auks, and allies plus buttonquails), Scolopaci (sandpipers, jacanas, and allies), and Charadrii (plovers, oystercatchers, and allies) [2]. This order is a monophiletic clade where the genus *Vanellus* from clade Charadrii is more basal than the genera *Tringa* and *Jacana* from clade Scolopaci [3]. Considering the great diversity in the number of species, shorebirds are an excellent model group to investigate several biological questions, such as morphology, ecological diversification, and phylogenetic relationships [4]. 

Regarding genome organization, the majority of reports on shorebirds are based on classical cytogenetics, limited in most cases to conventional staining with Giemsa (reviewed in Degrandi et al. [5]). However, these studies revealed that shorebirds have an exceptional range of diploid numbers, ranging from 2n = 42 in *Burhinus oedicnemus* [6] to 2n = 98 in *Gallinago gallinago* [7], indicating that interchromosomal rearrangements played important role in the evolutionary history of this group. Hence, considering that the typical avian karyotype has approximately 2n = 80 chromosomes [8], shorebirds represent an excellent model for studying chromosome evolution.

The first detailed studies focused on chromosome organization among shorebirds came from chromosome painting data using different sets of paints: *Gallus gallus* [6,9], *B*. *oedicnemus* [6,10,11,12], *Leucopternis albicollis* [9], and *Zenaida auriculata* [13]. These studies revealed extensive chromosome reorganization in some species, while others retained a conserved karyotype, similar to the putative avian ancestral karyotype. For instance, in *B*. *oedicnemus* (Clade Charadrii), chromosome reorganization involved mainly chromosome fusions [6], while in *Jacana jacana* (Clade Scolopaci), the process was mediated by chromosome fusions and fissions [13]. In *Actitis macularius* (Clade Scolopaci), several fissions involving macrochromosomes were described [11]. In *Larus argentatus* (Clade Lari), only fusions of macrochromosomes with microchromosomes were detected [10]. On the other hand, *Charadrius collaris* and *Vanellus chilensis*, both included in the Clade Charadrii, have a typical avian karyotype [9,12]. 

The data obtained from chromosome painting contributed to our knowledge about chromosome evolution in shorebirds; however, they were limited to the macrochromosomes in most of the reports (this is still true of most avian karyotype studies). Although *B*. *oedicnemus* provides insights about rearrangements involving microchromosomes, the exact role of these small elements in the rearrangements were not identified [10,11,12]. An alternative approach to investigate the microchromosome organization is bacterial artificial chromosomes (BACs) derived from chicken and zebra finch. These probes have been used in several avian orders, but interchromosomal rearrangements involving the microchromosomes were found only in a few avian orders [14,15,16,17,18]. Among shorebirds, BAC FISH is limited to *Scolopax rusticola* (Clade Scolopaci), where no evidence of interchromosomal rearrangements involving microchromosomes was found [16].

The presence of so many microchromosomes is a peculiar characteristic for nearly all birds. This feature possibly evolved around 250 million years ago [19,20]. The avian karyotype is characterized by containing around 2n = 80, among those 40 pairs, 30 pairs are usually microchromosomes with size ranging between 0.5 and 2.5 µm [21]. Some studies suggest that the common ancestor of birds presented microchromosomes in its karyotype, which possibly arose from chromosome fissions [22,23]. The presence of these tiny elements in the bird genome for such a long period of time implies an evolutionary success of these vertebrates [20,22,23].

Considering that information on cross-species chromosome mapping in shorebirds is limited to macrochromosomes, further studies are necessary to improve our understanding of the role of microchromosomes in the karyotype organization of these birds. Hence, in this study, we explored the microchromosome organization in three shorebirds species using chicken and zebra finch BACs. The aim was to improve our knowledge of its karyotype, especially regarding microchromosomes. Our results revealed a different pattern of microchromosome organization in each investigated species. We also performed a comparative analysis with related Charadriiformes and other birds.

## 2. Materials and Methods

### 2.1. Animals’ Collection and Chromosome Preparation

Samples (Table 1) were collected from individuals in their natural environment according to the permission of SISBIO 61047-4–ICMBio and the experiments were approved by the ethics committee from Universidade Federal do Pampa (CEUA 019/2020). For each individual, skin biopsies or feather pulp samples were collected to establish fibroblast cell culture in order to obtain chromosome preparations. Cells were cultured in flasks (25 cm^2^) with DMEM cell culture media (GIBCO), supplemented with 15% fetal bovine serum (GIBCO) and 1% penicillin (10,000 units/mL)/streptomycin (10,000 μg/mL) (GIBCO), and incubated at 37 °C [24]. Metaphase chromosomes were obtained according to standard procedures involving exposure to colcemid (1 h, 37 °C), hypotonic treatment (0.075 M KCl, 15 min, 37 °C), and fixation with methanol/acetic acid (3:1). *V. chilensis* species was also sampled by the direct chromosome preparation method, where embryo cells were dissociated by 2 mL of trypsin 0.25% EDTA for approximately 10 min, then the sample was placed in 10 mL of RPMI 1040 medium pre warmed at 37 °C with three drops of colchicine 0.05% and incubated for 1 h at 37 °C, followed by hypotonic treatment and fixation [25].

### 2.2. Karyotype Description

After chromosome harvesting, the cell suspension was dropped onto clean glass slides and air-dried, following the staining with Giemsa 5% in phosphate buffer with pH 6.8. To determine the diploid chromosome number and chromosomal morphologies, we analyzed at least 30 metaphases. Chromosomal morphology and karyotype ordering were determined according to Guerra [26].

### 2.3. FISH Experiments Using Chicken and Zebra Finch Bacterial Artificial Chromosomes (BACs)

Two BAC probes from chicken (*Gallus gallus*, CH261) or zebra finch (*Taeniopygia guttata*, TGMCBA) per microchromosomes (GGA10–28, except GGA16) were applied for cross-species FISH mapping in *Calidris canutus*, *Jacana jacana*, and *Vanellus chilensis* (Supplementary Materials Table S1). The BAC clone isolation, amplification, labeling, and hybridization were performed following the protocol described by O’Connor et al. [16]. The FISH results were confirmed by analyzing at least 10 metaphases per experiment. Images were captured with a CCD camera and SmartCapture (Digital Scientific UK) system, coupled to an Olympus BX61 epifluorescence microscope. Final image processing was performed using Adobe Photoshop 7.0.

Regardless of the fact that we used BAC probes from chicken and zebra finch libraries, the results were compared with chicken, once it represents the ancestral state. Most of the BAC probes used were obtained from chicken, but, for some microchromosomes, the chicken BACs did not hybridize successfully in all bird species [27]; in these cases, we used BAC probes from the zebra finch. In order to identify the chromosomal rearrangements, we considered the following: (i) no rearrangements if both BAC probes for each microchromosome produced FISH signals in the same microchromosome and with a size of micro; (ii) fission event when both BAC probes for each microchromosome produced FISH signals in different microchromosome; and (iii) fusion event when probes intended for a microchromosome hybridized to a macrochromosome.

## 3. Results

### 3.1. Karyotype Description

**Figure 1:**
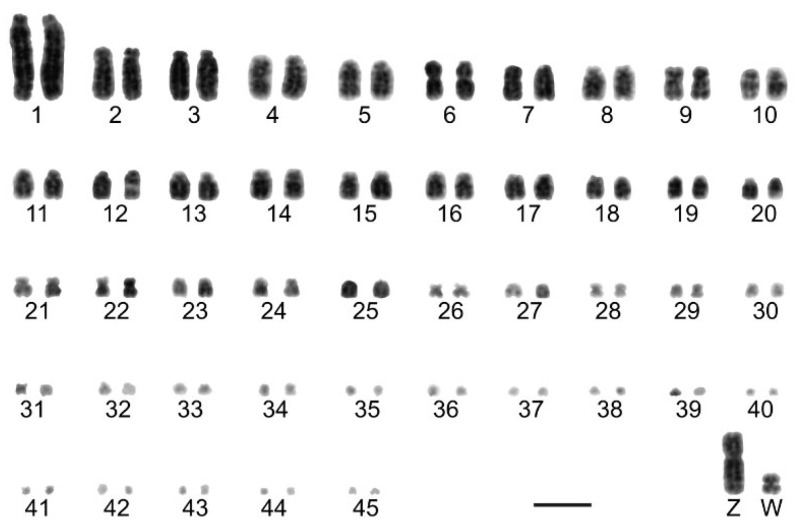
Conventionally stained complete karyotypes of *Calidris canutus* with 2n = 92. Bar 5 µm.

The karyotype of *Jacana jacana* (2n = 82) and *Vanellus chilensis* (2n = 78) was found to be consistent with previous studies [9,13]. However, we found a diploid number for *Calidris canutus* (2n = 92), which was different to 2n = 90, as previously described [28]. Our results showed that most autosomes are acrocentric, except for pairs 6 and 9, which are metacentric and submetacentric, respectively. The smallest microchromosomes are telocentric, the Z sex chromosome is a submetacentric macrochromosome with the size between the first and the second pairs, and the W sex chromosome corresponds to a small metacentric element with a size between pairs 20 and 21 (Figure 1).

### 3.2. Fluorescence In Situ Hybridization (FISH) Experiments

**Figure 2:**
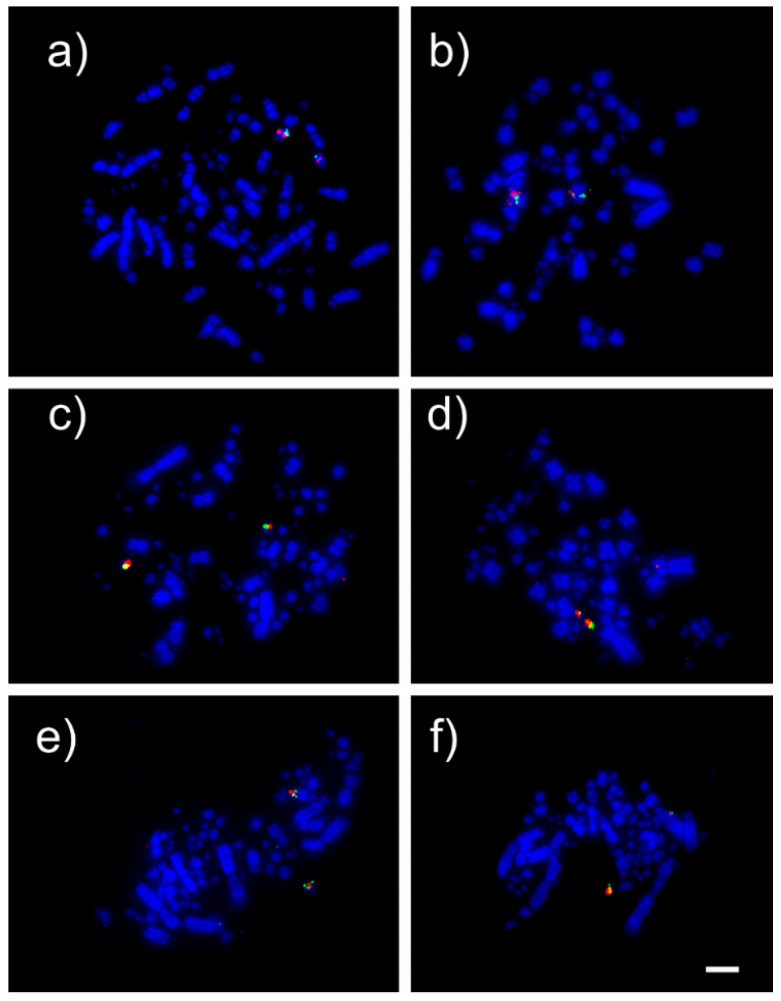
Examples of FISH experiments using chicken (GGA) bacterial artificial chromosome (BAC) probes in shorebirds. FISH results in *C. canutus*: chromosome 12 CH261-60P3 (red) and CH261-4M5 (green) (**a**) and chromosome 14 CH261-122H14 (red) and CH261-69D20 (green) (**b**). FISH results in *J. jacana*: chromosome 18 CH261-72B18 (red) and CH261-60N6 (green) (**c**) and chromosome 26 CH261-170L23 (red) and CH261-186M13 (green) (**d**). FISH results in *V. chilensis*: chromosome 17 CH261-42P16 (ged) and TGMCBA-375I5 (green) (e) and chromosome 28 CH261-72A10 (red) and CH261-64A15 (green) (**f**). Scale bar 5 µm.

The BAC FISH revealed different patterns of microchromosome organization in the species investigated. In *Calidris canutus*, two microchromosomes were involved in chromosome fusions (GGA 12 and GGA14). GGA12 probes produced signals in a medium macrochromosome, indicating the fusion with other microchromosome or a segment from a macrochromosome (as a result of fission events). GGA 14 hybridized on a macrochromosome, indicating a fusion of this chromosome with a macrochromosome. In *Jacana jacana*, the results revealed the conservation of the microchromosomes tested as one individual pair each; however, a gap in pair 8, previously described by Kretschmer et al. [13], remained unresolved, indicating that smaller chicken chromosomes were involved in that fusion. In contrast, there was no evidence of rearrangements involving microchromosomes in *Vanellus chilensis* (Figure 2). Table 2 summarizes the BAC FISH results in the three shorebirds investigated.

## 4. Discussion

BAC probes from chicken and zebra finch microchromosomes are a powerful tool to delineate chromosome homologies and to identify chromosomal rearrangements. This study thus contributed to the understanding of microchromosomes organization and evolution in shorebirds by investigating the karyotypes of *Calidris canutus*, *Jacana jacana*, and *Vanellus chilensis*. The karyotypes of *J*. *jacana* and *V*. *chilensis* have been previously described as 2n = 82 and 2n = 78, respectively [9,13], which was confirmed by our results. In addition, we found a new diploid number for *C*. *canutus*. It was previously described as 2n = 90 [28], but we found 2n = 92, owing to an additional pair of microchromosomes; however, the discrepancy between the new diploid number is probably due to technics limitations, which is a common mistake in avian classic cytogenetics because of its high number of very small microchromosomes.

Our molecular cytogenetic characterization using BAC FISH from the microchromosomes of chicken and zebra finch on metaphases of three shorebirds species confirmed that most of the chicken microchromosomes are conserved as entire units, as already reported in previous studies that demonstrated the high degree of conservation of microchromosomes in birds [16,29]. Interestingly, each species investigated here illustrated different microchromosome involvements in rearrangements. For instance, we found evidence of their involvement in interchromosomal rearrangements in *C*. *canutus* and *J*. *jacana*, while in *V*. *chilensis*, they were conserved as single pairs. In our study, we did not investigate the intrachromosomal rearrangements, as we used only two BAC probes per microchromosome. Nevertheless, these rearrangements in microchromosomes are very important features in bird genome owing to its capability of generating phenotypic differentiation, as reported for *Calidris pugnax*, where different mating phenotypes are described due to an intrachromosomal rearrangement on microchromosome 11 [30,31].

Analyzing the karyotype of *V*. *chilensis* and *C*. *collaris*, Pinheiro et al. [12] found differences in the microchromosomes with FISH signals using *Burhinus oedicnemus* probes corresponding to microchromosomes. These authors suggested that the variation in the number of signals was indicative of the considerable number of rearrangements involving microchromosomes in *V*. *chilensis*. However, our results disregard interchromosomal rearrangements involving the microchromosomes in this species. It is likely that the misinterpretation of FISH results by these authors was because of the background signal produced as a result of hybridization to repetitive sequences.

Although we did not find fusions involving microchromosomes in *J*. *jacana*, a previous study revealed a gap in pair 8 of this species, which was proposed as the result of a fusion between a microchromosome and a segment from the ancestral chromosome 5 (GGA5) [13]. This fusion remained unresolved, once none of the probes used in our study produced signals in the chromosome 8 of *J*. *jacana*. Nevertheless, it is important to highlight the fact that BAC probes corresponding to the chicken chromosome 28–39 have not been developed so far. Hence, a plausible explanation still relies on a possible fusion of a microchromosome pair within this range (pairs 28–39). 

In contrast, in *C*. *canutus,* microchromosome pairs 12 and 14 were involved in fusions. According to Kretschmer et al. [17], these microchromosomes are more likely to undergo interchromosomal rearrangements in birds. Fusion patterns differ between lineages, as observed in Waters et al. [29]. A possible explanation for the fusion events in *C*. *canutus* could be the presence and location of some specific motifs of repetitive sequence insertions, such as transposable elements, as observed in Psittaciformes species [32]. Besides that, no evidence of the occurrence of fissions of microchromosomes was observed in our results, indicating that the increase in the diploid number in *C*. *canutus* was due to macrochromosome fissions or even a smaller microchromosome fission (microchromosomes between 28–39). Similar results were observed in *Scolopax rusticola*, which have 2n = 96, and only macrochromosome fissions were found [16,33]. However, in *S*. *rusticola*, no evidence of microchromosome fusions was found.

BAC probes from microchromosomes have been used in several bird orders and have significantly contributed to our knowledge about microchromosome organization and evolution [14,16,17,18,27,34,35]. Interchromosomal rearrangements involving these tiny chromosomes were found only in some orders, such as Falconiformes, Psittaciformes, Caprimulgiformes, Cuculiformes, Suliformes, and Passeriformes, always in species with a relatively low diploid number for birds (usually lower than 2n = 74), indicating that the decrease in the diploid number was due to microchromosome fusions. However, to the best of our knowledge, this is the first time that fusions involving microchromosomes were found in a species with a high diploid number (e.g., *C*. *canutus*, 2n = 92), indicating that this type of rearrangements is not limited to species with a low diploid number.

Until now, including our study, the microchromosomal dynamics in karyotype evolution have been investigated in detail in four shorebirds species, three from the clade Scolopaci, *S*. *rusticola* [16], *C*. *canutus*, and *J*. *jacana*, and one from the clade Charadrii, *V*. *chilensis*. Considering that no interchromosomal rearrangements involving the microchromosomes were found in *S*. *rusticola*, we propose that the common ancestor for the clade Scolopaci had the ancestral pattern of microchromosome organization similar to *G*. *gallus*. After the divergence, each Scolopaci species has undergone different strategies in the microchromosome organization; that is, remained conserved as in *S*. *rusticola* or rearranged as in *C*. *canutus* and *J*. *jacana*. Similarly, the common ancestor for the clade Charadrii had the ancestral pattern once the microchromosome organization remained highly conserved in *V*. *chilensis*. 

Previously, analyzing the macrochromosomes using chromosome painting, we proposed that, after divergence, each shorebird suborder underwent different chromosome rearrangements [13], which was later confirmed by others [12]. Here, we extended this hypothesis to microchromosomes as well.

## 5. Conclusions

Our results illustrate that homology mapping using BAC probes for microchromosomes is necessary to understand the dynamics of genome reorganization in birds. The results of chromosome painting for both macro and microchromosomes of shorebirds suggest that the karyotypical evolution of these birds involved different chromosomal strategies in each clade. It is also important to highlight that, although the species are closely related, we have found different microchromosome behavior for each shorebird. Furthermore, our results in *Calidris canutus* indicated that species with a high diploid number could also undergo microchromosomal fusions.

## Figures and Tables

**Table 1:** List of the avian species investigated and the approaches used. Brazilian States: RS, Rio Grande do Sul; PA, Pará.

Species	Sex	Locality	Macrochromosomes Study	Microchromosomes Study
*Calidris canutus*	Female	Belém, PA, Brazil	-	Present study
*Jacana jacana*	Female	São Gabriel, RS, Brazil	Kretschmer et al. [13]	Present study
*Vanellus chilensis*	Male	São Gabriel, RS, Brazil	Kretschmer et al. [9]; Pinheiro et al. [12]	Present study

**Table 2:** Microchromosome correspondence between chicken and three shorebirds: *Vanellus chilensis* (VCH), *Jacana jacana* (JJA), and *Calidris canutus* (CCA).

Chicken Chromosomes	Species		
	VCH	JJA	CCA
GGA10 *	9	12	Micro
GGA11	11	16	Micro
GGA12	12	17	Fusion
GGA13	13	18	Micro
GGA14	14	19	Fusion
GGA15	15	20	Micro
GGA16	No data	No data	No data
GGA17	17	22	Micro
GGA18	18	23	Micro
GGA19	19	24	Micro
GGA20	20	25	Micro
GGA21	21	26	Micro
GGA22	22	27	Micro
GGA23	23	28	Micro
GGA24	24	29	Micro
GGA25	25	30	Micro
GGA26	26	31	Micro
GGA27	27	32	Micro
GGA28	28	33	Micro

* The chromosomal correspondence to GGA10 of *V*. *chilensis* (VCH) and *J*. *jacana* from Kretschmer et al. [9] and Kretschmer et al. [13], respectively.

## Data Availability

Not applicable.

## Supplementary Materials

The following supporting information can be downloaded at: https://www.mdpi.com/article/10.3390/ani12213052/s1, Table S1: List of BACs applied to shorebird species.
